# Acupoint Injection of Autologous Stromal Vascular Fraction and Allogeneic Adipose-Derived Stem Cells to Treat Hip Dysplasia in Dogs

**DOI:** 10.1155/2014/391274

**Published:** 2014-08-11

**Authors:** Camila Marx, Maiele Dornelles Silveira, Isabel Selbach, Ariel Silveira da Silva, Luisa Maria Gomes de Macedo Braga, Melissa Camassola, Nance Beyer Nardi

**Affiliations:** ^1^Laboratory of Stem Cells and Tissue Engineering, Universidade Luterana do Brasil, 92425-900 Canoas, RS, Brazil; ^2^Clinivetis Clinica Veterinaria, 93310-500 Novo Hamburgo, RS, Brazil; ^3^Center for Experimental Biological Models, Pontificia Universidade Catolica do Rio Grande do Sul, 90619-900 Porto Alegre, RS, Brazil

## Abstract

Stem cells isolated from adipose tissue show great therapeutic potential in veterinary medicine, but some points such as the use of fresh or cultured cells and route of administration need better knowledge. This study aimed to evaluate the effect of autologous stromal vascular fraction (SVF, *n* = 4) or allogeneic cultured adipose-derived stem cells (ASCs, *n* = 5) injected into acupuncture points in dogs with hip dysplasia and weak response to drug therapy. Canine ASCs have proliferation and differentiation potential similar to ASCs from other species. After the first week of treatment, clinical evaluation showed marked improvement compared with baseline results in all patients treated with autologous SVF and three of the dogs treated with allogeneic ASCs. On days 15 and 30, all dogs showed improvement in range of motion, lameness at trot, and pain on manipulation of the joints, except for one ASC-treated patient. Positive results were more clearly seen in the SVF-treated group. These results show that autologous SVF or allogeneic ASCs can be safely used in acupoint injection for treating hip dysplasia in dogs and represent an important therapeutic alternative for this type of pathology. Further studies are necessary to assess a possible advantage of SVF cells in treating joint diseases.

## 1. Introduction

Hip dysplasia (HD), characterized by instability and luxation or subluxation of hips, is an inherited orthopedic pathology that affects dogs of all breeds with different prevalences, causing pain and lameness. Treatment can be conservative, based on the restriction of exercise and administration of analgesics or chondroprotective agents, or surgical such as corrective osteotomy, pectineal myectomy, and lengthening of the femoral neck. These treatments demand too long to exert beneficial effects or are extremely invasive. More recently, cell therapy with mesenchymal stem cells (MSCs) was proposed as an alternative for this condition.

MSCs are primordial mesodermal cells present in all tissues and able to differentiate in vitro and in vivo in different cell types [1]. Their therapeutic potential is mainly explained by the production of bioactive molecules that provide a regenerative microenvironment in injured tissues [2]. The population of MSCs isolated from the adipose tissue (ASCs) has received special attention, due to their ease of collection, abundance, and regenerative potential [3]. Minimal criteria to define these cells, both as fresh stromal vascular fraction (SVF) and as cultivated ASCs, have recently been proposed by the International Federation of Adipose Therapeutics and International Society for Cellular Therapy [4].

Adipose-derived stem cells have been employed in veterinary medicine, mainly for bone, tendon, and ligament injuries and joint diseases. Canine MSCs isolated from the bone marrow, umbilical cord blood, adipose tissue, and Wharton's jelly have been characterized [5–9] or tested for therapeutic potential in canine models [10]. However, despite the increased use of stem cells in veterinary clinics [11], few studies have adequately investigated the efficacy of cell therapy [4, 12, 13]. Furthermore, some points should be better explored, such as the use of fresh cells from the SVF or cultured ASCs, number of cells used, and route of administration.

In this study, we propose the use of acupuncture points for stem cell therapy. The practice of acupuncture is very old in human medicine and is also a well-described veterinary procedure [14, 15], although some controversial opinions have been expressed [16]. Acupuncture has been used to treat dogs and cats for around 10 years, mainly for musculoskeletal problems [14], low back pain [17], knee osteoarthritis [18], tension-type headache, and migraine [19, 20]. Interestingly, acupuncture has been shown to mobilize stem cell compartments such as CD133+34− cells [21].

Our study aimed to evaluate the efficacy of autologous SVF or allogeneic cultivated ASCs injected into acupuncture points in dogs with HD.

## 2. Materials and Methods

### 2.1. Animals

The study protocol was approved by the Animal Use Ethics Committee of Universidade Luterana do Brasil (number 2010-1P). Adipose tissue was collected from three healthy male dogs of unknown breed, weighting on average 13.5 kg, maintained at the Animal House of the Veterinary Hospital of Universidade Luterana do Brasil. Canine patients in this study included three male and six female dogs of different breed, with HD and weak respond to drug therapy. Before inclusion, the owners agreed by informed consent to participate in the clinical study and to observe their dogs during the study period. The animals underwent routine clinical and hematological evaluation to ensure overall health, and any previous lesion was assessed by clinical and X-ray examination. A veterinarian assessed the dogs for pain on manipulation, alteration of range of motion, and functional disability.

### 2.2. Isolation of the Vascular Stromal Fraction

Adipose tissue (5–10 g) was collected from the inguinal region of four of the patients, using standard surgical procedures and mild anesthesia. The adipose tissue was washed with phosphate buffered saline (PBS) and digested with 1 mg/mL collagenase type I. After inactivation of the enzyme with an equal volume of Dulbecco's modified Eagle's Medium (DMEM) supplemented with 10% fetal calf serum (FCS, Cultilab, Sao Paulo, Brazil), the cells were washed with Hank's balanced salt solution and centrifuged at 400 ×g for 10 min. Viable cells in the SVF were counted after Trypan blue staining, and 5 × 10^6^ cells were resuspended in 2 mL PBS.

Unless otherwise stated, all reagents were from Sigma Chemical Co. (St. Louis, MO) and plasticware was from TPP (Trasadingen, Switzerland).

### 2.3. Isolation, Culture, and Characterization of ASCs

Adipose tissue (5–10 g) collected from the inguinal region of three healthy dogs was used for isolation of SVF and establishment of ASC cultures, as previously described [22]. Briefly, the cell pellet obtained after digestion with collagenase type I was resuspended in DMEM complemented with 2.5 g/L HEPES and 10% FCS and cultured in 5% CO_2_ at 37°C, with medium change every 2-3 days. Cells between passages 3 and 6 were used for characterization of the cultures, with analysis of 3 independent cultures, and for treatment of patients.

Cell proliferation was assessed by plating ASCs at 10^4^ cells/cm^2^, passaging at around 85% confluence at the same density, and counting recovered cells at every passage. Experiments were done in triplicate. The plasticity of ASC cultures was analyzed by incubation for up to 4 weeks with culture medium supplemented with specific factors for adipogenic, osteogenic, and chondrogenic lineages, as described [1, 22]. Adipocytes, osteoblasts, and chondrocytes were revealed with specific staining solutions (Oil Red O, Alizarin Red S, and Alcian Blue, resp.). All procedures had negative control cultures (undifferentiated cultures). Photomicrographs were taken with a digital camera (AxioCam MRc, Carl Zeiss, Oberkochen, Germany), using AxioVision 3.1 software (Carl Zeiss).

### 2.4. Treatments and Evaluation

Autologous cells of the vascular stromal fractions (from 2 to 5 × 10^6^ cells in 2 mL PBS) were used for treating four dogs. The remaining animals were treated with allogeneic, cultured adipose-derived stem cells (from 2 to 8 × 10^5^ cells in 2 mL PBS) derived from the same healthy donor dog. For the preparation of cell suspensions, viable cells were counted after Trypan blue staining. The cells were injected in three acupuncture points (bladder 54, gall bladder 29, and gall bladder 30) near the affected joint.

The animals were followed up in different periods by a veterinary, with physical and orthopedic examinations which assessed pain on manipulation, alteration of range of motion, and functional disability. Results were scored as worse, no modification, or improvement.

## 3. Results

**Figure 1:**
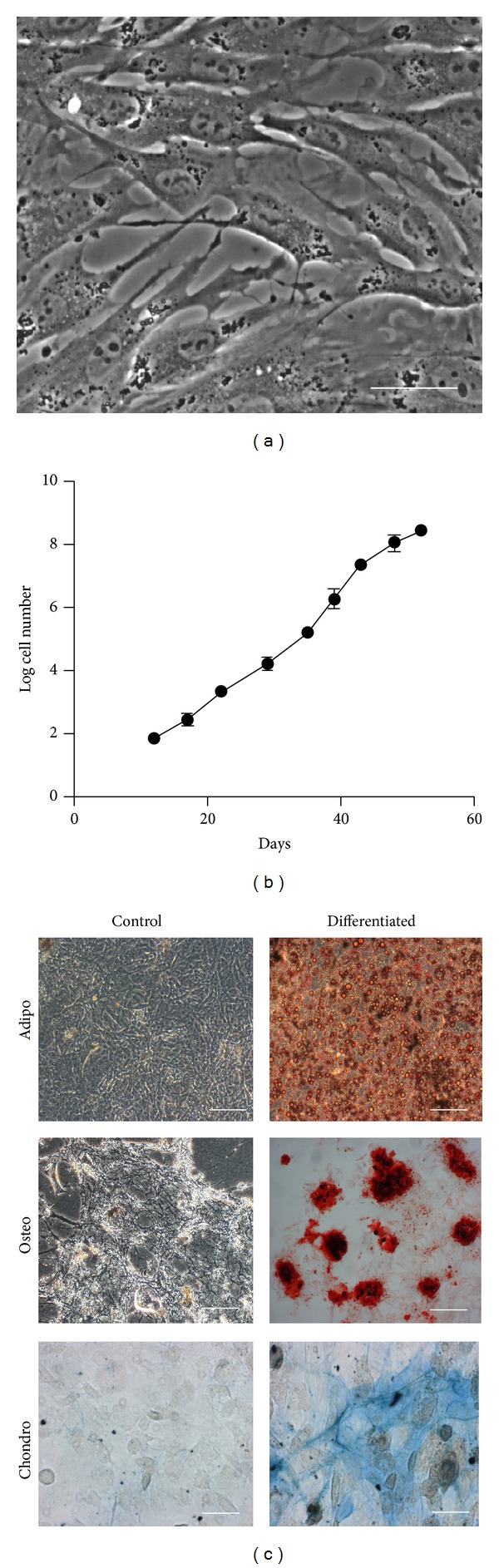
Morphology, proliferation, and differentiation potential of canine adipose-derived stem cells (ASCs). Cultures display the characteristic fibroblastoid morphology (a) and proliferation capacity (b) of ASCs from other species. ASCs are able to differentiate into adipogenic, osteogenic, and chondrogenic lineages, as shown by staining with Oil Red O, Alizarin Red S, and Alcian Blue, respectively (c). Undifferentiated (control) ASCs are not marked with these stains. Scale bars, 50 *μ*m ((a) and (c) Chondro) and 100 *μ*m ((c) Adipo, Osteo).

The cell yield in the vascular stromal fractions collected from the three normal dogs and four patients was on average 4.2 × 10^5^ ± 0.46 × 10^5^ viable cells/g of adipose tissue. For the patients in this study, specifically, the yield of viable cells in the SVF was 4.2 × 10^5^, 4.1 × 10^5^, 3.9 × 10^5^, and 5.1 × 10^5^, respectively, for patients from 6 to 9 shown in Table 1. Cell viability was higher than 95% in all isolation procedures. ASC cultures were successfully established from the SVF of the three normal donors. The cultures showed the typical morphology and proliferation rate of mesenchymal-type stem cells and were able to differentiate into adipocytes, osteoblasts, and chondrocytes (Figure 1).

The characteristics of the dogs included in this study, as well as the type of treatment received, are presented in Table 1. Injections through the acupuncture points were easily performed, without any sign of discomfort by the patients. The clinical outcomes are summarized in Table 2. In the first evaluation, conducted by the veterinarian one week after treatment, a clear improvement in range of motion and lameness at the trot and mainly a marked decrease in pain on manipulation were observed in seven of the patients. In dogs numbers 1 and 5, a less marked improvement was seen. According to the owners assessment, outcomes also improved markedly. Clinical evaluation on days 15 and 30 showed increased functional improvement in patients treated with autologous SVF (dogs from 6 to 9) and in three of the patients receiving allogeneic ASCs (dogs from 2 to 4). A recovery in range of motion and lameness was also observed in patient number 1 on day 15 and maintained on day 30. Patient number 5, however, showed difficulty in standing on day 15 and a complication of concomitant pathologies on day 30.

## 4. Discussion

The yield of viable cells in the SVF in our sample was similar to results previously reported [23]. Canine ASCs show morphology and proliferation and differentiation potential similar to ASCs from other species [24]. The proliferation rate of ASCs was not modified during culture period of at least 9 passages, so that cells used in passages from 3 to 6 were similar in this aspect. Immunophenotyping, one of the parameters to identify ASCs [4], was not included in the present study. Since antibodies against stem cells from canines and other species are not widely available, this parameter is not described in many of the studies published with stem cells from this species [12, 25, 26].

Allogeneic ASCs and autologous SVF showed similar therapeutic potential in hip dysplasia in dogs, when administered in acupuncture points. In only one of the dogs (patient number 5), a clear improvement was not observed. The complication of concomitant pathologies observed in this animal has not been related to cell therapy protocols [13]. The positive effect of stem cell therapy was more evident in patients treated with autologous SVF, but a larger sample number is necessary to establish the significance of this finding. The dose of SVF cells was planned according to a previous similar study [12], and since stem cells in the stromal vascular fraction range from 1% to 10% [4], ASC doses were correspondingly lower.

Many studies have assessed the therapeutic potential of the SVF in dogs, even though the terminology used to describe the cells has not been very clear. Black and colleagues [12, 27], for instance, showed that autologous SVF (which they named as “adipose-derived mesenchymal stem cell”) was effective to treat chronic osteoarthritis (OA) in dogs. Cultured ASCs have also been shown to have an important therapeutic activity in dogs. The clinical effect of a single injection of autologous, cultured ASCs was evaluated on 4 dogs with lameness associated with OA of the humeroradial joints [24]. OA of the elbow joints improved after treatment, suggesting a significant potential for the treatment of lameness. Similarly, Malik and colleagues [28] isolated and characterized canine ASCs, which were then successfully used to treat HD and paraplegic patients. Dogs recovered well and were able to move freely one month after treatment.

Few studies have compared the therapeutic potential of cultured cells and freshly isolated SVF or its equivalent in the bone marrow, the mononuclear fraction. In a study similar to ours, the therapeutic potential of fresh or cultured cells from the bone marrow mononuclear fraction in orthopedic lesions in dogs was compared [25]. Clinical ultrasound and X-ray showed beneficial results from both types of preparations, and the authors conclude that the use of fresh cells is advantageous since the technique is easier, the costs are lower, the surgery can be performed the same day, and the cells do not need to be expanded and preserve their initial potential. We agree only with this last consideration. The use of allogeneic, cultured cells does not require an additional surgery for collecting adipose tissue from the patient, and the use of an off-the-shelf product is faster and can be cheaper. Furthermore, advancing age of the donor has been shown to have a negative effect on the frequency and function of canine MSCs [23, 26], as already known for human cells [29]. However, it is possible that autologous, fresh cells are more efficient in repairing injured tissues, as suggested by the small difference seen in the two groups of animals in the present study.

The site of injection is an important variable to determine the results of cell therapy. The intra-articular administration of autologous ASCs in eight lame dogs with severe OA was measured with a force platform, which showed a significant increase in peak vertical force and vertical impulse after 6 months. Our results show that acupuncture points represent a potential administration route for stem cells. A long-term study involving 73 dogs with hip OA showed the efficacy of gold bead implantation inserted through needles at acupuncture points as a pain-relieving treatment and improved mobility [30]. This study supports the use of acupuncture for the treatment of HD and OA. Similarly, in a study including rats with hind limb ischemia, the injection of bone marrow mesenchymal stem cells in acupoint similar to the ones used in the present study improved the blood flow and increased the levels of vascular endothelial growth factor, transfer growth factor-B1, and nitric oxide, improving angiogenesis and arteriogenesis [31].

Future work is necessary to investigate the mechanisms responsible for the therapeutic effect of acupoint injection of these mesenchymal-type stem cells. MSCs are known to be able to migrate to sites of injury [32], and their therapeutic potential is more generally explained by a paracrine mechanism, with secretion of therapeutic bioactive factors [2], which could explain the results observed in the present study. A mere analgesic effect of the treatment, which could also explain some of the outcomes, would not be maintained for several weeks.

Our study indicates that adipose-derived stem cells, prepared as autologous SVF fresh cells or allogeneic cultured ASCs, can be safely used in acupoint injection for treating hip dysplasia in dogs and represent an important therapeutic alternative for this type of pathology. Further studies with a larger number of animals are necessary to assess the efficacy and a possible advantage of autologous fresh cells in treating joint diseases in dogs.

## Figures and Tables

**Table 1:** Characteristics of dogs included in the study and treatment received. Treatment and type of cells used.

#	Pathology	Breed	Gender	Age (years)	Treatment
1	G3 bilateral hip dysplasia + arthrosis and calcification of intervertebral discs^1^	GS	Female	9	ASCs
2	G3 bilateral hip dysplasia	GS	Female	10	ASCs
3	G2 left and G3 right hip dysplasia + arthrosis	LR	Female	6	ASCs
4	Bilateral G2 hip dysplasia + arthrosis	GS	Female	6	ASCs
5	G2 bilateral hip dysplasia	GS	Male	12	ASCs
6	Left G3 hip dysplasia + recent acetabular fracture	ACD	Male	0.5	SVF
7	G3 left and G2 right hip dysplasia	GR	Male	1	SVF
8	G1 left and G2 right hip dysplasia	GS	Female	8	SVF
9	G2 left and G1 right hip dysplasia	GS	Female	8	SVF

^1^Small degree of impact on usual activities

ACD: Australian Cattle Dog; ASCs: allogeneic adipose-derived cultured stem cells; G1: femoral head subluxated one quarter way out of the acetabulum; G2: femoral head subluxated half way out of the acetabulum; G3: femoral head subluxated three quarters way out of the acetabulum; GR: Golden Retriever; GS: German Shepherd; LR: Labrador Retriever; SVF: autologous stromal vascular fraction.

**Table 2:** Clinical outcomes regarding range of motion, lameness, and pain on manipulation on days 7, 15, and 30 after treatment with adipose-derived stem cells (ASCs, dogs numbers from 1 to 5) or autologous stromal vascular fraction (SVF, dogs numbers from 6 to 9).

Day	Dogs numbers 1–5 (ASCs)	Dogs numbers 6–9 (SVF)
7	Clear improvement in three patients (numbers from 2 to 4), particularly in pain on manipulation.	Clear improvement in all patients.
15	Improvement in four patients (numbers from 1 to 4).	Improvement maintained or increased in all patients.
30	Improvement maintained in four patients (numbers from 1 to 4).	Improvement maintained in all patients.
